# Health-related quality of life among people with diabetes: A cross-sectional study in Hail region, Saudi Arabia  ​

**DOI:** 10.1371/journal.pone.0299995

**Published:** 2024-05-07

**Authors:** Farhan Alshammari, Mukhtar Ansari, Kashif Ullah Khan, Dinesh Neupane, Arshad Hussain, Sirajudheen Anwar, Bushra Alshammari, Awatif Alrasheeday, Shazia Jamshed, Binaya Sapkota, Abdur Rasheed

**Affiliations:** 1 Department of Pharmaceutics, College of Pharmacy, University of Hail, Hail, Saudi Arabia; 2 Department of Clinical Pharmacy, College of Pharmacy, University of Hail, Hail, Saudi Arabia; 3 Department of International Health, Bloomberg School of Public Health, Johns Hopkins University, Baltimore, MD, United States of America; 4 Department of Pharmacology and Toxicology, College of Pharmacy, University of Hail, Hail, Saudi Arabia; 5 Medical Surgical Nursing Department, College of Nursing, University of Hail, Hail, Saudi Arabia; 6 Nursing Administration Department, College of Nursing, University of Hail, Hail, Saudi Arabia; 7 Pharmacy Practice, School of Pharmacy, International Medical University, Kuala Lumpur, Malaysia; 8 Jeffrey Sachs Center (JSC) on Sustainable Development, Sunway University, Selangor, Malaysia; 9 School of Public Health, Dow University of Health Sciences, Karachi, Pakistan; SPHMMC: St Paul’s Hospital Millennium Medical College, ETHIOPIA

## Abstract

**Background:**

Diabetes Mellitus is a serious and expanding health problem, together with the issues of health- related quality of life (HRQoL). This further puts pressure on the government to allocate more funds for public healthcare.

**Objectives:**

This study was devised to evaluate the health-related quality of life of people living with diabetes in Hail region of Saudi Arabia.

**Methods:**

This cross-sectional research was carried out at eight locations in the Hail region of Saudi Arabia between 21^st^ March-20^th^ May 2022 using the adapted version of the Euro QoL-5 dimension (EQ-5D-3L) questionnaire. A multistage random sample approach was used to choose the diabetes clinics, and data collectors approached the participants in the waiting areas to collect the information. The data were analyzed using logistic regression analysis, Mann-Whitney test, and Kruskal-Wallis tests in IBM SPSS statistics 21.0.

**Results:**

The mean HRQoL score was 0.71±0.21 with a visual analog score of 68.4±16.2. Despite having much higher levels of quality of life in terms of self-care (85.8%), regular activity (73.8%) and anxiety (71.8%), nearly one half of the people reported moderate pain or discomfort, and more than one third reported having moderate mobility issues. In general, the quality of life for women was poorer than for men. Individuals with diabetes who were unmarried, young, educated, financially secure, and taking only oral medication had much improved HRQoL. The Euro QoL of people with diabetes patients were significantly influenced by gender, marital status, age, education, employment and treatment modality (p-values < 0.05), whereas only treatment modality had a significant impact on the patients’ visual analogue measures (p-values < 0.05).

**Conclusions:**

The HRQoL of people with diabetes in Hail region was moderate in general, with pain and mobility issues being particularly prevalent. Gender, marital status, age, education, employment and type of medication therapy are significant predictors of HRQoL of patients with diabetes. Hence, planning and programs to enhance the HRQoL of people with diabetes, especially women is recommended.

## Introduction

Diabetes mellitus-type 2, which accounts for 90% of all cases and is on the rise, is a common chronic disease worldwide [1, 2]. Diabetes mellitus is a major and expanding health burden in Saudi Arabia as well, owing to prolonged physical distraction, hazardous food consumption, and increased obesity or body weight concerns [3, 4]. Saudi Arabia, a country in the Middle East and North Africa (MENA) region, has 18.7% prevalence of diabetes cases. By 2045, the MENA region would have the second-highest number of diabetes in the world with 136 million, with a regional prevalence rate of 16.2% and an anticipated increase of 86% [5].

Diabetes mellitus has several long-term complications if the glycemic management is not in the appropriate range. Heart attacks (i.e., myocardial infarction), strokes, kidney failure, blindness, and deformities of the lower limbs can all result from uncontrolled and untreated diabetes mellitus [6]. Additionally, it might result in reduced life expectancy, early mortality, and unemployment because of disabilities [7, 8]. To improve the health status, diabetes individuals must therefore maintain their wellbeing [9]. In addition to having a negative influence on individual morbidity and mortality rates, uncontrolled diabetes also compels the government to allocate more budget for national healthcare [10]. Hence, it is prudent to assess the health-related quality of life (HRQoL) of people with diabetes in the region in order to prioritize the problem and enable policymakers and program implementers to implement effective programs to enhance the same.

## Methods

### Study setting and design

The Hail region of Saudi Arabia was selected for this cross-sectional study, and the study was carried out between 21^st^ March-20^th^ May 2022. The most important healthcare providers for people with diabetes in each chosen location were determined to be referral or tertiary care hospitals (including diabetes clinics or diabetes care centers). Hail region is one of the thirteen regions or provinces of Saudi Arabia and is in the northwest with a population of 731,147 as of 2019 [11]. Hatim al-Tai, a legendary figure who represents generosity and curiosity, hails from this area.

### Study population

Males and females with type 2 diabetes from the Hail region of Saudi Arabia were the subjects of the study. Participants in the study had to be at least 18 years old, have been diagnosed with type 2 diabetes for more than a year, to be able to comprehend and speak Arabic and/or English, and provide their consent to participate. However, the study excluded women recently diagnosed with type 2 diabetes as well as women who were pregnant or had gestational diabetes. In a similar way, children and those with type 1 diabetes were excluded.

### Study tool

The adapted Euro QoL-5 dimension three-level (EQ-5D-3L) in conjunction with the Euro QoL visual analog scale (EQ-VAS) health questionnaire served as the study tool. The EQ-5D-3L health questionnaire was used in this study because it is reliable and valid, has better responsiveness among people with diabetes, and is user-friendly [12–14]. The EQ-5D’s first section asks patients to self-report their current health condition in relation to five different categories: mobility, self-care, daily activities, pain or discomfort, and anxiety or depression. There are three possible answers for each dimension: no problems, some or moderate problems, and severe problems. For every question, there is a scale that ranges from 1 (no problem) to 3 (severe problem). The score digits were used to generate five-digit codes (EQ-5D codes) representing each patient’s HRQoL, i.e., 11111 as the best possible score, and 33333 the worst possible score. The EQ-5D codes in the first section of the EQ-5D health questionnaire were further transformed into a single weighted index score (EQ-5D_index_) using a United Kingdom scoring algorithm) [15–17]. The United Kingdom scoring system has been used to the Saudi population in the past and is frequently utilized in situations where country-specific weights are unavailable [18]. The second part of the EQ-5D uses a visual analogue scale (VAS) with values ranging from 0 to 100 to gauge the subjects’ perception of their quality of life (QoL); 0 represents the worst possible state, and 100 represents the best possible health status (EQ-VAS).

As the study participants were Saudis, Arabic adaptation of the surveys was utilized to get precise responses. The World Health Organization (WHO) benchmark was used for translating the questionnaires from English to Arabic [19]. The process of forward and backward translation was used to validate the translated version of the study tools.

### Sample size and sampling procedure

According to the International Diabetes Federation (IDF), 18.7 percent of Saudi Arabian adults had diabetes in 2021 [5]. A minimum required sample size of 232 was calculated using the prevalence-based formula (n = Z^2^*P(1-P)/d^2^), where n is the required sample size, P denotes the disease prevalence (18.5 percent, P = 0.18), Z = confidence level (95 percent, corresponding to a standard value of 1.96), and d = margin of error (standard value of 0.05).

The study subjects were sampled using a multistage random sampling method, a probability sampling approach. In the first step, four geographical areas of Hail were chosen at random. The second step consisted of a random selection of two hospitals from each area with diabetes clinics, followed by a random selection of patients. Every alternate patient seated in the diabetes clinic’s seats was chosen using a systematic random sampling process. While the patients wait for their turn, data collector met them in the waiting areas of the diabetes clinics and explained the study objectives and details to them. After the participants gave their written consents, they were provided with a study information sheet and a copy of the questionnaires (EQ-5D). For patients who could not read or write, the patient’s first-degree relative or a companion filled out the questionnaire. Authors had no access to information that could identify individual participants during or after data collection.

### Validation of questionnaire

The study tool was piloted using 15 individuals and its reliability was evaluated using Cronbach’s alpha, which obtained a value of 0.87 (good reliability). Academics, epidemiologists, and healthcare professionals made up a team of experts who evaluated the study tool’s face and content validity.

### Data management and analysis

The data set was imported into IBM SPSS statistics 21.0, and analyzed for descriptive and inferential analysis. After looking at the skewness of the data, it was found that the data were not normally distributed. Thus, non-parametric tests were used to analyze the data. There were five domains of health state, i.e., mobility, self-care, usual activities, pain/discomfort, and anxiety/depression. The outcomes of each domain were in three categories such as; no problem, some/mild problem and extreme/severe problem. In terms of mobility, for instance, the three options were ‘no problems in walking’, ‘some problems in walking’ and ‘confined to bed’. Similarly, for self-care, the three options were ‘no problems with self-care’, ‘some problems washing or dressing myself’, and ‘unable to wash or dress myself’. As with usual activities, the responses were ‘no problems with performing my usual activities’, ‘some problems with performing my usual activities’ and ‘unable to perform my usual activities’. Similarly, for pain/discomfort, the responses were ‘no pain or discomfort’, ‘moderate pain or discomfort’ and ‘extreme pain or discomfort’. Finally, the three categories identified for the anxiety/depression were ‘not anxious or depressed’, ‘moderately anxious or depressed’ and ‘extremely anxious or depressed’. In order to apply binary logistic regression, each domain’s outcomes were converted from three to two categories. For example, mobility results were split into two categories-, no problem and any problem while walking. Similarly, self-care results were split into, no problem and any problem in washing or dressing, usual activities results in, no problem and any problem, pain/discomfort results in no pain and any pain, and anxiety/depression results in, no anxiety/depression and any anxiety/depression. Following the bivariate logistic regression analysis, multivariable logistic regression was also performed, but only on the variables that had previously been shown to be significant (p-value < 0.05).

Similarly, the quality of life and visual analogue scale were compared across patient demographics using the Mann–Whitney and Kruskal–Wallis tests. The differences were considered significant if the p-value remained below 0.05.

### Ethical considerations and approval

Each piece of information remained private and anonymous. Data was also completely encoded and coded for usage with computer applications, primarily for statistical analyses. The Saudi Arabian Ministry of Health granted ethical approval for this study with a registration number of H-08-L-074.

## Results

The study included 400 participants with 50.5% (n = 202) women, and mean age±SD of 53±9.4 years. Almost three-quarters of the participants in the study (74%) were married, and more than half (55.8%) of the participants were ≥50 years of age. Even though many participants were educated, 19% of them did not have any formal education. The unemployed and retired made up nearly 60% of the participants. Moreover, the monthly income for about one-third of the patients (36%) was less than 5000 Saudi Riyals, and over 56% of the patients were only using oral antidiabetic medication (Table 1).

**Table 1:** Patients’ demographic variables (n = 400).

Variables		Frequency	Percent
Gender	Male	198	49.5
	Female	202	50.5
Marital status	Single	49	12.2
	Married	296	74
	Widowed	31	7.8
	Divorced	24	6
Age (years)	18–29	32	8
	30–39	48	12
	40–49	97	24.25
	50–59	109	27.25
	60–69	80	20
	≥70	34	8.5
Education Level	No formal education	76	19
	Primary	66	16.5
	Secondary	90	22.5
	Technical degree	69	17.2
	Bachelors	87	21.8
	Higher education	12	3
Employment status	Employed	156	39
	Unemployed	163	40.7
	Retired	75	18.8
	Unable to work due to health problem	6	1.5
Monthly income (SAR)	Less than 5000 (i.e., <1332.91 USD)	144	36
	5000–9,999 (i.e., 1332.91–2665.55 USD)	100	25
	10,000–19,999 (i.e., 2665.82–5331.36 USD)	142	35.5
	20,000 and above (i.e., >5331.63 USD)	14	3.5
Type of treatment	Oral antidiabetic medicine only	226	56.5
	Insulin only	70	17.5
	Oral antidiabetic medicine and insulin combination	104	26

**Note**: SAR-Saudi Arabian Riyal (1 SAR = 0.27 USD as per the conversion rate of December 2, 2023)

**Fig 1:**
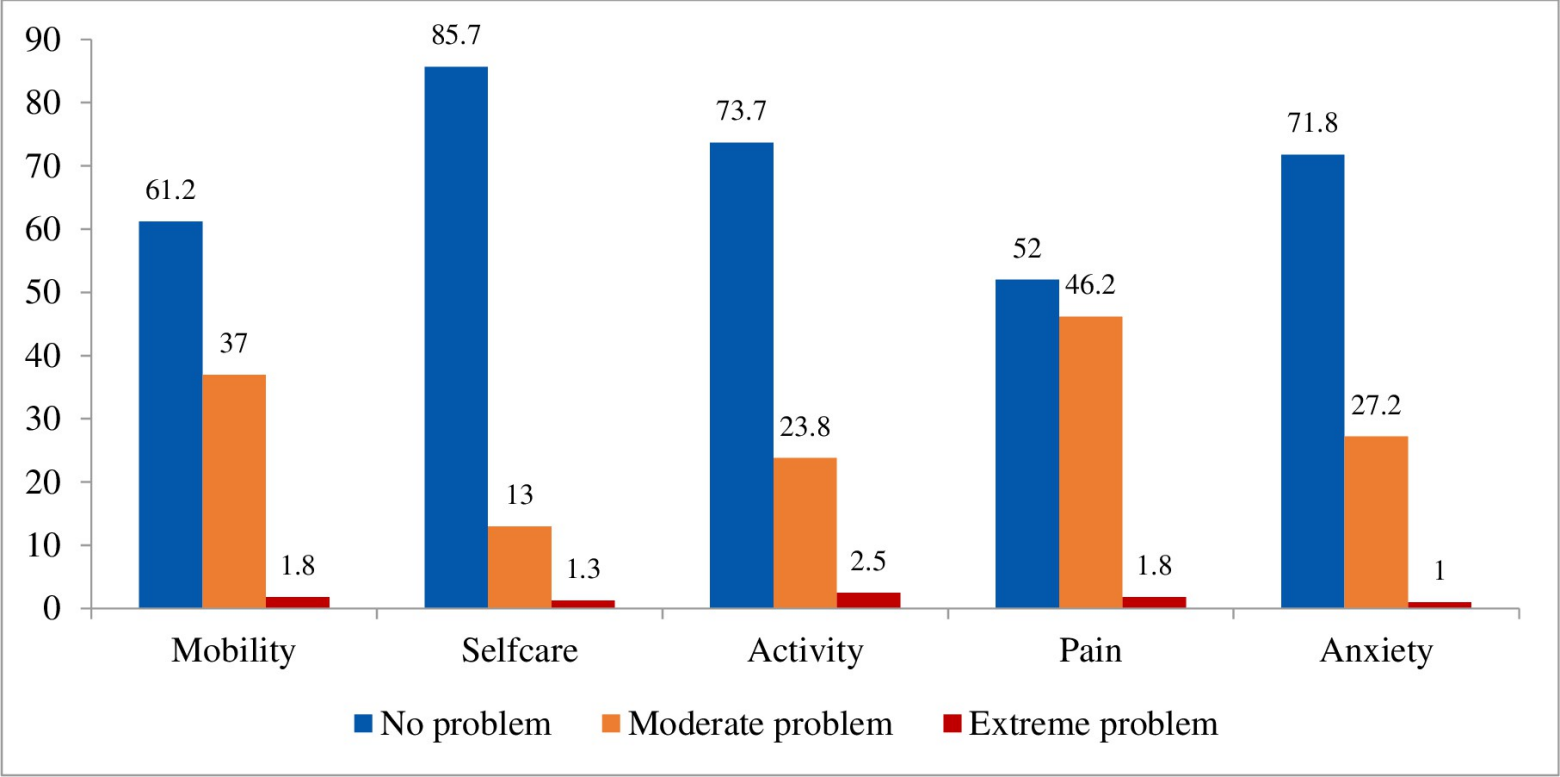
Health problem in terms of EQ-5D-3L. Blue color stands for ‘no problem’, orange color represents ‘moderate problem’, and red color indicates ‘extreme problem’.

Despite having much higher levels of quality of life in terms of self-care (85.7%), regular activity (73.7%) and anxiety (71.8%), nearly one half of the people with diabetes experienced moderate pain or discomfort, and over one third reported having moderate mobility issue. However, compared to other dimensions of health status, usual activity, mobility and pain were more adversely affected by severe or extreme problems (Fig 1).

Table 2 shows both bivariate and multivariable logistic regression analyses. Since all variables in the bivariate analysis were significant, the multivariable logistic regression analysis was carried out. The adjusted odds ratio and 95% CI were mentioned following the adjustment of all the independent variables presented in Table 2. It was observed that the only variables that remained significant were age, education, and type of treatment. After adjusting for other variables, multivariable logistic regression revealed that the likelihood of experiencing a problem while walking increased with age. For example, patients in the 40–49 age group were 10.36 times more likely to experience a problem while walking than patients in the 18–29 age group, and this likelihood increased to 50.02 for patients in the 60–69 age group. After adjusting all other variables, patients with a bachelor’s degree were 86% less likely to experience problem while walking as compared to patients without any formal education. This indicates a declining trend in the likelihood of problems with higher education (Table 2).

**Table 2:** Logistic regression analysis among demographic variables and mobility (n = 400).

Variables	Mobility		Mobility (no problem = 245, any problem = 155)					
			Bivariate			Multivariable		
	No problem	Any problem	COR	P-value	(95% CI)	AOR	P-value	(95% CI)
**Gender**				<0.001*			0.607	
Male	138	60	1			1		
Female	107	95	2.042	<0.001*	(1.35, 3.07)	1.21	0.607	(0.59, 2.46)
**Marital status**				<0.001*			0.249	
Single	40	9	1			1		
Married	186	110	2.628	0.013*	(1.23, 5.62)	0.55	0.268	(0.19, 1.59)
Widowed	6	25	18.519	<0.001*	(5.88, 58.34)	1.5	0.599	(0.33, 6.81)
Divorced	13	11	3.761	0.016*	(1.28, 11.08)	0.61	0.485	(0.15, 2.47)
**Age group, (years)**				<0.001*			0.001*	
18–29 years	31	1	1			1		
30–39 years	39	9	7.154	0.069	(0.86, 59.55)	8.63	0.068	(0.85, 87.13)
40–49 years	73	24	10.192	0.026*	(1.32, 78.70)	10.36	0.046*	(1.04, 103.2)
50–59 years	60	49	25.317	0.002*	(3.34, 192.14)	20.93	0.01*	(2.09, 209.97)
60–69 years	31	49	49	<0.001*	(6.36, 377.4)	50.02	0.001*	(4.85, 516.0)
≥70 years	11	23	64.818	<0.001*	(7.8, 538.38)	23.62	0.012*	(2.01, 278.11)
**Education Level**				<0.001*			0.001*	
No formal education	18	58	1			1		
Primary	28	38	0.42	0.019*	(0.21, 0.87)	0.63	0.263	(0.28, 1.41)
Secondary	65	25	0.12	<0.001*	(0.06, 0.24)	0.19	<0.001*	(0.08, 0.45)
Technical degree	50	19	0.12	<0.001*	(0.06, 0.25)	0.27	0.011*	(0.10, 0.74)
Bachelors	74	13	0.05	<0.001*	(0.02, 0.12)	0.14	<0.001*	(0.05, 0.4)
Higher education	10	2	0.06	0.001*	(0.01, 0.31)	0.18	0.066	(0.03, 1.12)
**Employment level**				<0.001*			0.061	
Employed	125	31	1			1		
Unemployed	76	87	4.62	<0.001*	(2.8, 7.61)	1.88	0.157	(0.78, 4.51)
Retired	43	32	3	<0.001*	(1.64, 5.49)	1.01	0.988	(0.43, 2.34)
Unable to work	1	5	20.16	0.007*	(2.27, 178.84)	21.16	0.017*	(1.72, 260.15)
**Monthly income (SAR)**				0.001*			0.575	
Less than 5000	73	71	1			1		
5000–9,999	57	43	0.78	0.332	(0.46, 1.30)	1.59	0.22	(0.76, 3.35)
10,000–19,999	104	38	0.38	<0.001*	(0.23, 0.62)	1.7	0.208	(0.75, 3.87)
20,000 and above	11	3	0.28	0.059	(0.08, 1.05)	1.9	0.419	(0.4, 9.08)
**Type of treatment**				<0.001*			0.008*	
Only oral medication	153	73	1			1		
Only insulin	47	23	1.03	0.931	(0.58, 1.82)	0.92	0.827	(0.43, 1.96)
Oral medication and insulin combination	45	59	2.75	<0.001*	(1.7, 4.43)	2.47	0.003*	(1.36, 4.49)

Table 3 represents bivariate and multivariable logistic regression analysis for usual activities. Since all variables in the bivariate analysis were significant, the multivariable logistic regression analysis was carried out. After adjustment of all the independent variables presented in Table 3, adjusted odds ratio and 95% CI were mentioned. It was observed that only education and type of treatment variables remained significant. Multivariable logistic regression showed that, following adjustment for all other variables, there was a trend toward a decreasing likelihood of problem in usual activities with respect to higher education (except primary). For example, patients with a bachelor’s degree were 74% less likely to experience problem in usual activities as compared to patients with no any formal education. Furthermore, after adjusting all variables, patients with insulin were 0.06 times less likely to have problem, while patients with insulin and oral medication had 2.23 times higher chances of experiencing problems in their usual activities.

**Table 3:** Logistic regression analysis among demographic variables and usual activities (n = 400).

Variables	Usual activities		Usual activities (no problem = 295, any problem = 105)					
			Bivariate			Multivariable		
	No problem	Any problem	COR	P-value	(95% CI)	AOR	P-value	(95% CI)
**Gender**				<0.001*			0.402	
Male	164	34	1			1		
Female	131	71	2.61	<0.001*	(1.64, 4.18)	1.39	0.402	(0.64, 3.02)
**Marital status**				<0.001*			0.333	
Single	42	7	1			1		
Married	224	72	1.93	0.127	(0.83, 4.48)	1	0.997	(0.3, 3.3)
Widowed	11	20	10.91	<0.001*	(3.68, 32.35)	2.28	0.279	(0.51, 10.2)
Divorced	18	6	2	0.266	(0.59, 6.79)	0.74	0.707	(0.15, 3.57)
**Age group, (years)**				<0.001*			0.105	
18–29 years	28	4	1			1		
30–39 years	43	5	0.81	0.773	(0.2, 3.3)	0.81	0.812	(0.15, 4.46)
40–49 years	78	19	1.71	0.368	(0.53, 5.45)	1.13	0.884	(0.22, 5.73)
50–59 years	80	29	2.54	0.106	(0.82, 7.86)	1.34	0.723	(0.26, 6.87)
60–69 years	54	26	3.37	0.038*	(1.07, 10.62)	2.09	0.391	(0.39, 11.28)
≥70 years	12	22	12.83	<0.001*	(3.63, 45.33)	5.31	0.079	(0.83, 34.21)
**Education Level**				<0.001*			0.013*	
No formal education	37	39	1			1		
Primary	36	30	0.79	0.486	(0.41, 1.53)	1.53	0.279	(0.71, 3.28)
Secondary	73	17	0.22	<0.001*	(0.11, 0.44)	0.46	0.074	(0.2, 1.08)
Technical degree	59	10	0.16	<0.001*	(0.07, 0.36)	0.41	0.106	(0.14, 1.21)
Bachelors	79	8	0.1	<0.001*	(0.04, 0.23)	0.26	0.017*	(0.09, 0.79)
Higher education	11	1	0.09	0.022*	(0.01, 0.7)	0.24	0.229	(0.02, 2.47)
**Employment level**				<0.001*			0.285	
Employed	137	19	1			1		
Unemployed	96	67	0.14	0.02*	(0.03, 0.74)	1.94	0.164	(0.76, 4.9)
Retired	59	16	0.7	0.665	(0.14, 3.56)	0.91	0.853	(0.33, 2.49)
Unable to work	3	3	0.27	0.131	(0.05, 1.47)	4.47	0.217	(0.41, 48.18)
**Monthly income (SAR)**				0.001*			0.173	
Less than 5000	89	55	1			1		
5000–9,999	79	21	0.43	0.005*	(0.24, 0.77)	0.73	0.407	(0.35, 1.53)
10,000–19,999	115	27	0.43	<0.001*	(0.22, 0.65)	1.79	0.167	(0.78, 4.11)
20,000 and above	12	2	0.43	0.094	(0.06, 1.25)	2.33	0.346	(0.4, 13.6)
**Type of treatment**				<0.001*			0.021*	
Only oral medication	180	46	1			1		
Only insulin	55	15	1.07	0.846	(0.55, 2.06)	0.94	0.887	(0.42, 2.11)
Oral medication and insulin combination	60	44	2.87	<0.001*	(1.73, 4.76)	2.23	0.009*	(1.22, 4.07)

Table 4 represents bivariate and multivariable logistic regression analysis for pain/discomfort. Once again, all variables were adjusted for multivariable logistic regression analysis, and only education and type of treatment variables were found significant. The chances of experiencing pain/discomfort diminishes with higher education. When all the other variables were adjusted, diabetic patients who completed their graduation had 71% less chances of experiencing pain/discomfort, and those who had a higher level of education had 85% less chances as compared to patients who had no formal education.

**Table 4:** Logistic regression analysis among demographic variables and pain/discomfort (n = 400).

Variables	Pain/discomfort		Pain/discomfort (no pain = 208, any pain = 192)					
			Bivariate			Multivariable		
	No pain	Any pain	COR	P-value	(95% CI)	AOR	P-value	(95% CI)
**Gender**				<0.001*			0.329	
Male	124	74	1			1		
Female	84	118	2.35	<0.001*	(1.58, 3.52)	1.35	0.329	(0.74, 2.49)
**Marital status**				<0.001*			0.278	
Single	34	15	1			1		
Married	158	138	1.98	0.039*	(1.04, 3.79)	0.75	0.534	(0.3, 1.87)
Widowed	6	25	9.44	<0.001*	(3.21, 27.77)	2.14	0.294	(0.52, 8.83)
Divorced	10	14	3.17	0.026*	(1.15, 8.75)	0.97	0.962	(0.27, 3.5)
**Age group, (years)**				<0.001*			0.313	
18–29 years	26	6	1			1		
30–39 years	32	16	2.17	0.157	(0.74, 6.33)	2.44	0.191	(0.64, 9.25)
40–49 years	56	41	3.17	0.02*	(1.2, 8.41)	2.66	0.15	(0.7, 10.05)
50–59 years	49	60	5.31	0.001*	(2.02, 13.92)	3.95	0.048*	(1.01, 15.4)
60–69 years	34	46	5.86	<0.001*	(2.17, 15.81)	4.36	0.041*	(1.06, 17.94)
≥70 years	11	23	9.06	<0.001*	(2.89, 28.39)	2.6	0.246	(0.52, 13.03)
**Education Level**				<0.001*			0.013*	
No formal education	17	59	1			1		
Primary	22	44	0.58	0.146	(0.27, 1.21)	0.87	0.742	(0.38, 1.98)
Secondary	53	37	0.2	<0.001*	(0.1, 0.4)	0.3	0.005*	(0.13, 0.7)
Technical degree	45	24	0.15	<0.001*	(0.07, 0.32)	0.28	0.009*	(0.11, 0.73)
Bachelors	61	26	0.12	<0.001*	(0.06, 0.25)	0.29	0.009*	(0.11, 0.73)
Higher education	10	2	0.06	0.001*	(0.01, 0.29)	0.15	0.035*	(0.02, 0.88)
**Employment level**				<0.001*			0.455	
Employed	104	52	1			1		
Unemployed	60	103	3.43	<0.001*	(2.17, 5.44)	1.59	0.253	(0.72, 3.53)
Retired	42	33	1.57	0.117	(0.89, 2.76)	0.97	0.948	(0.44, 2.15)
Unable to work	2	4	4	0.116	(0.71, 22.56)	3.53	0.242	(0.43, 29.24)
**Monthly income (SAR)**				0.003*			0.822	
Less than 5000	60	84	1			1		
5000–9,999	50	50	0.71	0.199	(0.43, 1.19)	1.34	0.417	(0.66, 2.69)
10,000–19,999	88	54	0.44	0.001*	(0.27, 0.7)	1.42	0.369	(0.66, 3.04)
20,000 and above	10	4	0.29	0.042*	(0.09, 0.95)	1.44	0.607	(0.36, 5.86)
**Type of treatment**				<0.001*			<0.001*	
Only oral medication	132	94	1			1		
Only insulin	46	24	0.73	0.276	(0.42, 1.28)	0.63	0.195	(0.32, 1.26)
Oral medication and insulin combination	30	74	3.46	<0.001*	(2.1, 5.71)	3.14	<0.001*	(1.78, 5.55)

Bivariate and multivariable logistic regression analysis for self-care is shown in Table 5. Given their significance in the bivariate analysis, gender, education, employment, and types of treatment variables were taken into consideration for the multivariable logistic regression analysis. The likelihood of experiencing self-care issues decreases steadily with higher education. Moreover, when adjustments for gender, education, employment were made, patients who used insulin in combination with oral medication were 4 times more likely to experience self-care issues.

**Table 5:** Logistic regression analysis among demographic variables and self-care (n = 400).

Variables	Self-care		Self-care (no problem = 343, any problem = 57)					
			Bivariate			Multivariable		
	No problem	Any problem	COR	P-value	(95% CI)	AOR	P-value	(95% CI)
**Gender**				0.004*			0.483	
Male	180	18	1			1		
Female	163	39	2.39	0.004*	(1.32, 4.35)	1.42	0.483	(0.53, 3.77)
**Marital status**								
Single	49	0						
Married	251	45						
Widowed	20	11	NA	NA	NA	NA	NA	NA
Divorced	23	1						
**Age group, (years)**								
18–29 years	32	0						
30–39 years	45	3						
40–49 years	92	5	NA	NA	NA	NA	NA	NA
50–59 years	89	20						
60–69 years	67	13						
≥70 years	18	16						
**Education Level**				<0.001*			0.008*	
No formal education	50	26	1			1		
Primary	54	12	0.43	0.034*	(0.19, 0.94)	0.48	0.09*	(0.21, 1.12)
Secondary	80	10	0.24	0.001*	(0.11, 0.54)	0.27	0.005*	(0.11, 0.68)
Technical degree	62	7	0.22	0.001*	(0.09, 0.54)	0.28	0.024*	(0.09, 0.84)
Bachelors	85	2	0.05	<0.001*	(0.01, 0.2)	0.06	0.001*	(0.01, 0.31)
Higher education	12	0	0	0.999	(0, 0)	0	0.999	(0, 0)
**Employment level**				0.001*			0.756	
Employed	146	10	1			1		
Unemployed	127	36	4.14	<0.001*	(1.97, 8.67)	1.06	0.909	(0.38, 2.95)
Retired	66	9	1.99	0.154	(0.77, 5.13)	1.32	0.626	(0.43, 4.09)
Unable to work	4	2	7.3	0.032*	(1.19, 44.8)	3.13	0.296	(0.37, 26.64)
**Monthly income (SAR)**				0.476				
Less than 5000	120	24	1			1		
5000–9,999	84	16	0.95	0.89	(0.48, 1.9)	-	-	
10,000–19,999	126	16	0.63	0.191	(0.32, 1.25)	-	-	
20,000 and above	13	1	0.38	0.368	(0.05, 3.08)	-	-	
**Type of treatment**				<0.001*			<0.001*	
Only oral medication	208	18	1			1		
Only insulin	63	7	1.28	0.593	(0.51, 3.21)	1.3	0.592	(0.5, 3.43)
Oral medication and insulin combination	72	32	5.14	<0.001*	(2.72, 9.71)	4.06	<0.001*	(2.06, 8.01)

It was not necessary to apply multivariable logistic regression for anxiety or depression since no variable in bivariate logistic regression analysis found significance (Table 6).

**Table 6:** Logistic regression analysis among demographic variables and anxiety/depression (n = 400).

Variables	Anxiety/depression		Anxiety/depression (no anxiety = 287, any anxiety = 113)		
			Bivariate		
	No anxiety	Any anxiety	COR	P-value	(95% CI)
**Gender**				0.515	
Male	145	53	1		
Female	142	60	1.16	0.515	(0.75, 1.79)
**Marital status**				0.945	
Single	35	14	1		
Married	213	83	0.97	0.939	(0.5, 1.9)
Widowed	21	10	1.19	0.726	(0.45, 3.16)
Divorced	18	6	0.83	0.748	(0.27, 2.54)
**Age group, (years)**				0.992	
18–29 years	22	10	1		
30–39 years	35	13	0.82	0.687	(0.31, 2.18)
40–49 years	68	29	0.94	0.885	(0.4, 2.23)
50–59 years	80	29	0.8	0.606	(0.34, 1.88)
50–59 years	58	22	0.83	0.692	(0.34, 2.04)
≥70 years	24	10	0.92	0.871	(0.32, 2.62)
**Education Level**				0.115	
No formal education	52	24	1		
Primary	45	21	1.01	0.976	(0.5, 2.05)
Secondary	70	20	0.62	0.175	(0.31, 1.24)
Technical degree	42	27	1.39	0.342	(0.7, 2.76)
Bachelors	68	19	0.61	0.161	(0.3, 1.22)
Higher education	10	2	0.43	0.304	(0.09, 2.13)
**Employment level**				0.918	
Employed	112	44	1		
Unemployed	115	48	1.06	0.807	(0.65, 1.73)
Retired	56	19	0.86	0.646	(0.46, 1.62)
Unable to work	4	2	1.27	0.785	(0.23, 7.2)
**Monthly income (SAR)**				0.837	
Less than 5000	100	44	1		
5000–9,999	72	28	0.88	0.667	(0.5, 1.55)
10,000–19,999	104	38	0.83	0.478	(0.5, 1.39)
20,000 and above	11	3	0.62	0.479	(0.17, 2.33)
**Type of treatment**				0.168	
Only oral medication	170	56	1		
Only insulin	45	25	1.69	0.075	(0.95, 3)
Oral medication and insulin combination	72	32	1.35	0.254	(0.81, 2.26)

Table 7 shows comparisons of EQ-5D and EQ-VAS with the study participants’ demographics. Mean, SD, medians, and Interquartile ranges are given, and p-values were obtained with nonparametric tests. It is evident that EQ-5D scores differed significantly among all demographic variables (p-values < 0.05). However, VAS did not differ significantly among demographic variables except for treatment variable where a significant median difference was noted.

**Table 7:** Comparisons of EQ-5D and EQ-VAS with the study participants’ demographics (n = 400).

Variables	n	EQ-5D Score					EQ-VAS Score				
		Mean	SD	Median	IQR	p-value	Mean	SD	Median	IQR	p-value
**Gender** ^a^						< 0.001*					0.491
Male	198	0.841	0.16	0.87	0.19		79.01	15.9	80	20	
Female	202	0.794	0.14	0.78	0.28		77.19	17.9	80	20	
**Marital status** ^b^						< 0.001*					0.718
Single	49	0.87	0.12	0.96	0.19		78.8	14.9	80	20	
Married	296	0.82	0.15	0.86	0.25		78.1	16.8	80.50	20.5	
Widowed	31	0.68	0.14	0.70	0.16		75.1	18.4	80	20.5	
Divorced	24	0.82	0.12	0.82	0.25		79.5	20.7	80	37.5	
**Age, (years)** ^b^						< 0.001*					0.405
18–29	32	0.90	0.08	0.96	0.1		79.0	14.3	80	20	
30–39	48	0.87	0.12	0.96	0.18		81.5	15.1	80	30	
40–49	97	0.85	0.12	0.87	0.19		75.5	18.1	80	25	
50–59	109	0.80	0.14	0.80	0.26		79.6	17.0	80	20	
60–69	80	0.78	0.13	0.77	0.1		78.1	15.2	80	20	
70 & above	34	0.66	0.23	0.67	0.33		74.4	21.2	80	22.5	
**Education Level** ^b^						< 0.001*					0.135
No formal education	76	0.72	0.13	0.70	0.17		75.3	18.7	70	20	
Primary	66	0.74	0.19	0.74	0.23		79.8	17.7	80	20	
Secondary	90	0.84	0.14	0.87	0.19		78.9	15.0	80	20	
Technical degree	69	0.84	0.12	0.86	0.19		79.4	14.3	80	20	
Bachelors	87	0.88	0.11	0.96	0.1		75.8	18.7	80	20	
Higher education	12	0.91	0.06	0.93	0.1		87.5	12.1	80	20	
**Employment level** ^b^						< 0.001*					0.078
Employed	156	0.86	0.12	0.89	0.18		78.1	16.3	80	20	
Unemployed	163	0.77	0.14	0.77	0.33		76.6	18.1	80	20	
Retired	75	0.81	0.18	0.86	0.24		80.0	16.6	80	20	
Unable to work	6	0.64	0.27	0.73	0.42		68.3	7.52	70	7.5	
**Treatment modality** ^b^						< 0.001*					0.007*
Only Oral medication	226	0.84	0.13	0.87	0.19		78.8	16.7	80	20	
Only Insulin	70	0.83	0.12	0.86	0.21		81.6	15.2	80	20	
Oral medication and insulin combination	104	0.73	0.18	0.77	0.24		74.1	18.0	70	15.2	

EQ-5D: EuroQoL-5 dimension; SD: standard deviation; VAS: visual analogue scales; IQR: interquartile range; HRQoL: health-related quality of life.

^a^Mann–Whitney test

^b^Kruskal–Wallis test

*p < 0.05.

## Discussion

The health-related quality of life offers a multifaceted perspective, and is a key consideration in managing one’s diabetes and minimizing the risk of diabetes-related complications. One of the factors contributing to lower HRQoL is the noticeable percentage (37.6%) of undiagnosed diabetes cases in MENA region [5]. In our study, the mean HRQoL score was 0.71±0.21. This overall HRQoL is the modest, which is consistent with a Jordanian study [20]. Despite the moderate overall HRQoL obtained in our study, 46.2% of people with diabetes rated pain to be one of their most bothersome symptoms. The mean HRQoL of women in our study was lower than that of men; moreover, women were more than twice as likely as men to experience pain and difficulty walking, engage in usual activities, and self-care. These findings concur with several studies [12, 21–23]. This is particularly true for women, who are more likely to have worse HRQoL because they spend considerable time at home taking care of their families and often struggle with severe anxiety and despair [24]. In contrast, a study from Oman indicated that women understood their diabetes better than men did, and that they had higher levels of HRQoL than men did perhaps because of their higher levels of physical activity [25].

Patients who were single or unmarried reported a considerably higher HRQoL than those who were married, divorced or widowed. In particular, these patients had almost double the risk of having trouble in walking, difficulties with usual activities and pain as compared to single individuals. Patients who are single or unmarried tend to have better HRQoL since they are less financially burdened for their family and do not have spouse commitments. This is in line with an Iranian study that found those who were single or unmarried had better quality of life [26]. Contrary to this, a study carried out in Riyadh, Saudi Arabia [18] found that married patients had much better HRQoL; this can be demonstrated by the fact that they receive additional family care.

In this study, majority of the patients with diabetes were 50 years of age or older. Elderly patients generally experienced more difficulty walking, performing usual activities and pain or discomfort, whereas patients over 70 years of age had upsurge rates of mobility. Studies have also shown that as people age, their HRQoL declines [27–29]. While the risk of diabetes-related anxiety or depression was notably lower in older patients in our study, research has shown that patients with diabetes often have comorbid illnesses, which exacerbate HRQoL [30].

Several studies [26, 31–33] have validated findings of this study, which showed that a higher level of education was frequently associated with a higher quality of life. This is particularly related to the idea that those with higher levels of education are more conscious of diseases and the complications that may arise due to ignorance or negligence. Higher educated male patients exhibited considerably higher HRQoL average scores, which align with a study by Zare et al. [26]. This is because men are often more physically active than women [34, 35].

A sizeable proportion of patients with diabetes in this study-approximately one-third-had monthly income below 5000 Saudi Riyals. While on the other side, there is a wider spectrum of necessities and household spending demands. The psychological strain that results from lesser income and increasing demand puts elderly and their caregivers at higher risk of developing health issues like diabetes. The finding is consistent with those of Gaskin et al., who found that people with lower socioeconomic status have a higher risk of developing diabetes-related complications, which lower HRQoL [36]. Higher monthly income earners in this study demonstrated a lower risk of mobility issues, pain or discomfort, and difficulties performing usual activities, self-care and anxiety. Higher monthly income generally led to an improved HRQoL, which is consistent with research findings from other studies [37–39].

Studies on the impact of insulin and oral hypoglycemic medications on quality of life have yielded mixed findings. A Turkish study by Akinci et al., found that patients on insulin had a better quality of life than those on oral hypoglycemic medications [40]. However, a study by Lingvay et al., reported no difference in QoL between patient groups on insulin and oral hypoglycemic medications [41]. Our study’s findings demonstrated that patients taking oral medication and insulin concurrently had considerably poorer HRQoL. This could be the result of insulin’s and oral hypoglycemic agents’ adverse effects on overall QoL [42].

This research does have some limitations. The study was carried out in one region of the country only that could not represent the entire nation. On the other hand, low frequencies were occasionally observed during analysis, which could result in wider 95% confidence intervals and possibly have an impact on the outcome due to the skewed distribution of some categories.

## Conclusions

The HRQoL of people with diabetes in Hail region was moderate in general, with pain and mobility issues being notably high. Gender, marital status, age, education level, employment and type of therapy are significant predictors of HRQoL of patients with diabetes. Therefore, it is crucial to develop measures to enhance the HRQoL of people with diabetes, particularly women.

We strongly recommend assessing the HRQoL of patients with diabetes in Saudi Arabia with a multi-center, preferably nationwide study involving bigger sample size.

## Supporting information

S1 Checklist STROBE statement—Checklist of items that should be included in reports of observational studies. (DOCX)
