# Supplementary material for: Health-related quality of life among people with diabetes: A cross-sectional study in Hail region, Saudi Arabia ​
Source: PLoS One. 2024 May 7;19(5):e0299995. doi: 10.1371/journal.pone.0299995 (PMC11075843; doi:10.1371/journal.pone.0299995)
Supplement: S1 Checklist — (DOCX) [file pone.0299995.s001.docx]

STROBE Statement—checklist of items that should be included in reports of observational studies

|  | Item No. | Recommendation | Page  No. | Relevant text from manuscript |
| --- | --- | --- | --- | --- |
| **Title and abstract** | 1 | (*a*) Indicate the study’s design with a commonly used term in the title or the abstract | 1 | Assessing diabetes patients' quality of life in relation to their health in Hail region Saudi Arabia::A multistage random sampling method |
|  |  | (*b*) Provide in the abstract an informative and balanced summary of what was done and what was found | 3 | This cross-sectional research was carried out at eight locations in the Hail region of Saudi Arabia between March-May 2022 using the adapted version of the Euro QoL-5 dimension (EQ-5D) questionnaire.  The mean health related quality of life score was 0.71 ± 0.21 with a Visual Analog Score of 68.4 ± 16.2. |
| Introduction | | | |  |
| Background/rationale | 2 | Explain the scientific background and rationale for the investigation being reported | 4 | Diabetes Mellitus is a major and expanding health burden in Saudi Arabia, owing to prolonged physical distraction, hazardous food consumption, and increased weight concerns.  Diabetes mellitus has a number of long-term complications if the glycemic management is not in the appropriate range. It might result in reduced life expectancy, early mortality, and unemployment as a result of disabilities |
| Objectives | 3 | State specific objectives, including any prespecified hypotheses | 4 | To assess the health-related quality of life of diabetes patients in the region in order to prioritize the problem and enable policymakers and program implementers to implement effective programs to enhance health related quality of life (HRQoL) of the people |
| Methods | | | |  |
| Study design | 4 | Present key elements of study design early in the paper | 5 | Cross-sectional study |
| Setting | 5 | Describe the setting, locations, and relevant dates, including periods of recruitment, exposure, follow-up, and data collection | 5 | The study was carried out between March-May 2022 in the Hail region of Saudi Arabia. Referral or tertiary care hospitals (including diabetes clinics or diabetes care centers) were chosen in each location as the most important healthcare provider for people with diabetes. |
| Participants | 6 | (*a*) *Cohort study*—Give the eligibility criteria, and the sources and methods of selection of participants. Describe methods of follow-up  *Case-control study*—Give the eligibility criteria, and the sources and methods of case ascertainment and control selection. Give the rationale for the choice of cases and controls  *Cross-sectional study*—Give the eligibility criteria, and the sources and methods of selection of participants | 5 | The study's participants were people with type 2 diabetes who were at least 18 years old, had been diagnosed with it for more than a year, could understand and speak Arabic and/or English, and had consented to participate. However, type 2 diabetes patients who had recently received their diagnosis as well as women who were pregnant and had either gestational diabetes or type 2 diabetes were not included in the study. Children and those with type 1 diabetes were also excluded in a similar manner.  The individuals were sampled using a multistage random sampling method, a probability sampling approach. In the first step, four geographical areas of Hail were chosen at random. The second step consisted of a random selection of two hospitals from each areas with diabetic clinics, followed by a random selection of patients. While the patients wait for their turn, data collector met them in the waiting areas of the diabetes clinics and explained the study to them. After the participants gave written consent, they were provided with study information sheet and a copy of the questionnaires (EQ-5D). For patients who couldn't read or write, the questionnaire was filled out by the patient's first-degree relative or a companion. |
|  |  | (*b*) *Cohort study*—For matched studies, give matching criteria and number of exposed and unexposed  *Case-control study*—For matched studies, give matching criteria and the number of controls per case | NA | NA |
| Variables | 7 | Clearly define all outcomes, exposures, predictors, potential confounders, and effect modifiers. Give diagnostic criteria, if applicable | 5-6 | The adapted Euro QoL-5dimension (EQ-5D) health questionnaire was used as the survey tool.  The Euro QoL (EQ) has a maximum score of 10 and a minimum score of zero, since the scores for EQ5D vary from 0 to 2. A score of zero in any health status domain means there are no issues, a score of one indicates a slight or moderate issue, and a score of two indicates an extreme or severe issue. |
| Data sources/ measurement | 8* | For each variable of interest, give sources of data and details of methods of assessment (measurement). Describe comparability of assessment methods if there is more than one group | 7 | The data was entered into IBM SPSS statistics 21.0, and assessed for descriptive and inferential analyses. Multivariate logistic regression analysis was employed to see the relationships between demographic variables and five domains of health state. Similarly, Mann–Whitney and Kruskal–Wallis tests were used to examine whether the visual analogue scale and quality of life were related to the demographics of diabetic patients. The associations were deemed significant as long as the p-value remained below 0.05 throughout the analyses. |
| Bias | 9 | Describe any efforts to address potential sources of bias | 6 | Authors had no access to information that could identify individual participants during or after data collection. |
| Study size | 10 | Explain how the study size was arrived at | 6 | According to the International Diabetes Federation (IDF), 18.7 percent of Saudi Arabian adults had diabetes in 2021. A minimum required sample size of 232 was calculated using the prevalence-based formula (n=Z^2^*P(1-P)/d^2^), where n is the required sample size, P denotes the disease prevalence (18.5 percent, P = 0.18), Z= confidence level (95 percent, corresponding to a standard value of 1.96), and d= margin of error (standard value of 0.05). |

Continued on next page

| Quantitative variables | 11 | Explain how quantitative variables were handled in the analyses. If applicable, describe which groupings were chosen and why | 7 | Multivariate logistic regression analysis was employed to see the relationships between demographic variables and five domains of health state. Similarly, Mann–Whitney and Kruskal–Wallis tests were used to examine whether the visual analogue scale and quality of life were related to the demographics of diabetic patients. |
| --- | --- | --- | --- | --- |
| Statistical methods | 12 | (*a*) Describe all statistical methods, including those used to control for confounding |  |  |
|  |  | (*b*) Describe any methods used to examine subgroups and interactions |  |  |
|  |  | (*c*) Explain how missing data were addressed |  |  |
|  |  | (*d*) *Cohort study*—If applicable, explain how loss to follow-up was addressed  *Case-control study*—If applicable, explain how matching of cases and controls was addressed  *Cross-sectional study*—If applicable, describe analytical methods taking account of sampling strategy | 7 | Multivariate logistic regression analysis was employed to see the relationships between demographic variables and five domains of health state. Similarly, Mann–Whitney and Kruskal–Wallis tests were used to examine whether the visual analogue scale and quality of life were related to the demographics of diabetic patients. |
|  |  | (*e*) Describe any sensitivity analyses |  |  |
| Results | | | | |
| Participants | 13* | (a) Report numbers of individuals at each stage of study—eg numbers potentially eligible, examined for eligibility, confirmed eligible, included in the study, completing follow-up, and analysed | 8 | Table 1 |
|  |  | (b) Give reasons for non-participation at each stage |  |  |
|  |  | (c) Consider use of a flow diagram |  |  |
| Descriptive data | 14* | (a) Give characteristics of study participants (eg demographic, clinical, social) and information on exposures and potential confounders | 8 | Table 1 |
|  |  | (b) Indicate number of participants with missing data for each variable of interest |  |  |
|  |  | (c) *Cohort study*—Summarise follow-up time (eg, average and total amount) |  |  |
| Outcome data | 15* | *Cohort study*—Report numbers of outcome events or summary measures over time |  |  |
|  |  | *Case-control study—*Report numbers in each exposure category, or summary measures of exposure |  |  |
|  |  | *Cross-sectional study—*Report numbers of outcome events or summary measures | 11-12 | Table 2, Table 3 |
| Main results | 16 | (*a*) Give unadjusted estimates and, if applicable, confounder-adjusted estimates and their precision (eg, 95% confidence interval). Make clear which confounders were adjusted for and why they were included | 11-12 | Table 2, Table 3 |
|  |  | (*b*) Report category boundaries when continuous variables were categorized |  |  |
|  |  | (*c*) If relevant, consider translating estimates of relative risk into absolute risk for a meaningful time period |  |  |

Continued on next page

| Other analyses | 17 | Report other analyses done—eg analyses of subgroups and interactions, and sensitivity analyses | 11-12 | Table 2, Table 3 |
| --- | --- | --- | --- | --- |
| Discussion | | | | |
| Key results | 18 | Summarise key results with reference to study objectives | 12-14 | In our study, the mean health related quality of life score was 0.71± 0.21.  Despite having much higher levels of quality of life in terms of self-care, regular activity and anxiety, people had annoying experiences in terms of pain, mobility and anxiety. |
| Limitations | 19 | Discuss limitations of the study, taking into account sources of potential bias or imprecision. Discuss both direction and magnitude of any potential bias | 14 | This research does have some limitations. The study was carried out in one region of the country only that could not represent the entire nation. Thus, the findings of this study highlight the necessity for a significant nationwide investigation to evaluate the level of health related quality of life of diabetic patients in Saudi Arabia |
| Interpretation | 20 | Give a cautious overall interpretation of results considering objectives, limitations, multiplicity of analyses, results from similar studies, and other relevant evidence | 15 | Although health related quality of life of people with diabetes in Hail region was moderate in general, people had annoying experiences in terms of pain, mobility and anxiety. Patients who were married had a superior quality of life in terms of mobility. Diabetes patients, particularly married women frequently experienced anxiety. In addition to gender, education level and type of therapy were significant EQ-5D-3L predictors in this study. Therefore, it is crucial to develop measures to enhance the HRQoL of people with diabetes, particularly women. |
| Generalisability | 21 | Discuss the generalisability (external validity) of the study results | 15 | In addition to gender, education level and type of therapy were significant EQ-5D-3L predictors in this study. |
| Other information | |  | | |
| Funding | 22 | Give the source of funding and the role of the funders for the present study and, if applicable, for the original study on which the present article is based | 15 | The Research Deanship of the University of Ha'il - Saudi Arabia provided funding for this study under grant number (RG-20204). The funding agency, however, had no influence over the design of study, collection of data, article preparation and publication choice. |

*Give information separately for cases and controls in case-control studies and, if applicable, for exposed and unexposed groups in cohort and cross-sectional studies.

**Note:** An Explanation and Elaboration article discusses each checklist item and gives methodological background and published examples of transparent reporting. The STROBE checklist is best used in conjunction with this article (freely available on the Web sites of PLoS Medicine at http://www.plosmedicine.org/, Annals of Internal Medicine at http://www.annals.org/, and Epidemiology at http://www.epidem.com/). Information on the STROBE Initiative is available at www.strobe-statement.org.
